# The Clustering of Health-Related Behaviors in the Adult Japanese Population

**DOI:** 10.2188/jea.JE20200120

**Published:** 2021-08-05

**Authors:** Claire Mawditt, Kiriko Sasayama, Kota Katanoda, Stuart Gilmour

**Affiliations:** 1 Canon Foundation in Europe, Amstelveen, Netherlands; 2 Graduate School of Public Health, St. Luke’s International University, OMURA Susumu & Mieko Memorial, St. Luke’s Center for Clinical Academia, Tokyo, Japan; 3 Division of Cancer Statistics Integration, Center for Cancer Control and Information Services, National Cancer Center, Tokyo, Japan

**Keywords:** health-related behavior, clustering, latent profile analysis, Japan, gender

## Abstract

**Background:**

Research findings indicate that four health-related behaviors (HRBs), smoking, alcohol, diet, and physical activity, do not co-occur within individuals by chance and therefore cluster. To date, there is a lack of research investigating the clustering of these HRBs in the Japanese population.

**Methods:**

The Japanese National Health and Nutrition Survey 2010 was used, containing information on 8,015 community-dwelling adults. Latent profile analysis identified distinct cluster patterns of four HRBs: smoking status, alcohol consumption, calorie intake, and the number of steps per day.

**Results:**

For men, four distinct HRB clusters were identified. The largest cluster (54%) was characterized by drinking more than Japan’s recommended alcohol guidelines and walking an inadequate number of steps per day. A small cluster (4%) also emerged, characterized by smoking, high calorie intake, and exceeding alcohol guidelines. Members of these clusters had higher systolic blood pressure than those in the remaining clusters. For women, five distinct HRB clusters were identified. The largest cluster (57%) was characterized by not smoking or drinking and walking an inadequate number of steps per day. For both genders, there was a relationship between cluster membership and age. Cluster membership was associated with income and health status among men but not women.

**Conclusion:**

Detecting distinct clusters of HRBs in a Japanese population-based survey provides a person-centered understanding of Japanese lifestyles. This approach can assist policy makers in Japan and overseas to identify new strategies for targeting behavioral risk factors and make health promotion policies more effective in their respective countries.

## INTRODUCTION

Research findings indicate that people practice multiple health-related behaviors (HRBs) in their everyday lives and that these HRBs do not co-occur within individuals by chance and therefore cluster.^1^^,^^2^ This suggests that HRBs are inter-related and that the clustering approach is necessary to study patterns of health behavior. Moreover, research indicates that HRB cluster membership is linked to age, gender, socio-economic position,^1^^,^^2^ and health status.^3^

These clusters may consist of negative HRBs that are health damaging (eg, smoking, heavy alcohol consumption, a diet high in sugar and fat and low in fruit and vegetables, and physical inactivity), positive HRBs that are health promoting (eg, not smoking, light or moderate alcohol consumption, a diet low in sugar and fat and high in fruit and vegetables and low in sugar and fat, and regular physical activity), or a mixture of both.

From a public health perspective, addressing negative HRBs and promoting positive HRBs is important because they are modifiable.^4^ However, many policies to address negative HRBs and promote positive HRBs are implemented in isolation and do not consider the dynamics of HRB clustering, instead considering them as individual and unrelated entities.^2^ By taking a more sophisticated approach and considering the inter-relatedness of these four HRBs, as well as associated socio-demographics and health status, we can gain a deeper understanding of the types of people who share HRB patterns, which can inform the development of lifestyle interventions that target specific subgroups of the population.^5^

The presence of multiple negative HRBs during mid-life increases the risk of developing non-communicable diseases (NCDs) in later life in Japan.^6^^–^^8^ However, to date there is a lack of research investigating the clustering of these four HRBs in a representative sample of the adult Japanese population.

Similar to the United Kingdom and other developed countries, Japan has an aging population and an increasing burden of NCDs.^9^ Therefore, identifying similar HRB cluster patterns in the Japanese population to those found in other developed countries can provide stronger evidence that shared associations may be generalized to other cohorts and countries, offering insights for policy makers internationally.

However, the distribution of HRBs in the Japanese population is different from other developed countries.^10^ Japan has a high prevalence of tobacco use in men,^11^ high levels of salt intake,^12^ and very low levels of overweight and obesity.^13^ Japan also has a very high life expectancy and is recognized as a global leader in health outcomes.^14^ This implies that the findings from research investigating the clustering of HRBs in other developed countries^1^^,^^2^ cannot necessarily be generalized to the Japanese context. Therefore, elucidating country differences may serve to generate new hypotheses as to how the unique experiences of the Japanese population may influence their lifestyles.

In this study, we conducted a detailed investigation of HRB clustering within the Japanese population. Detecting distinct clusters of HRBs in a Japanese population-based survey and their relationship with socio-demographic factors and health status will provide a person-centered understanding of Japanese lifestyles that can inform population-level interventions to improve HRBs among Japanese adults. Moreover, detecting Japanese HRB cluster patterns may offer lessons to other countries in strategies for improving behavioral risk factors and make their health promotion policies more effective.

## METHODS

### Data

The Japanese National Health and Nutrition Survey (NHNS) from 2010 was used.^15^^–^^17^ The NHNS is a nationally representative probabilistic sample of community-dwelling individuals in Japan, designed to collect basic data on their health, nutrition, and lifestyles. Individual level data were obtained via official application to the Ministry of Health, Labour and Welfare. Prior to data collection, an ethical standards committee gave approval for the conduct the study and trained interviewers visited each address to obtain informed consent from participants.

### Measures

Four HRBs were measured: smoking status, alcohol consumption, calorie intake, and physical activity.

Information on smoking and alcohol consumption was captured for individuals aged 20 years and over in the lifestyle component of the survey. Participants were asked about their smoking behavior. First, they were asked if they had smoked at least 100 cigarettes in their life. Second, they were asked if they smoke cigarettes daily, some days, or not at all. Current smokers were defined as having smoked at least 100 cigarettes in their life and smoke every day or some days, and former smokers were those who did not smoke at all presently but had previously smoked more than 100 cigarettes in their life. Non-smokers were those who had never smoked more than 100 cigarettes in their life.

Responses to questions regarding the quantity and frequency of alcohol consumption were used to derive a three-category variable measuring if participants drank any alcohol, and among those who did, whether their alcohol use met the current Japanese government’s alcohol consumption guidelines (ie, no more than 20 g alcohol per day for men or 10 g for all women and men aged 65 or older, 5 days or less per week).^18^

Total calorie intake was calculated via a semi-weighed and portion size recording method, which was completed alongside the lifestyle survey. A designated person in each household was trained by a professional to record the weight (or portion size if weighing proved difficult) of food before and/or after preparation, its distribution among household members, and any waste and/or leftovers on a designated day (excluding Sunday) in November.^16^ Standard Tables of Food Composition in Japan were used to convert records into nutrient and calorie intake.^15^ The physical activity measure was based upon the observed number of daily step counts captured via a pedometer worn by participants aged 15 years or older on the same day as the dietary intake questionnaire. Upon completion, trained interviewers reviewed the questionnaires with each household representative to check and clarify their entries.

Two socio-demographic variables, age and income, were considered as covariates in this study. Individuals under 20 years old were excluded due to a lack of information on certain HRBs (ie, smoking and alcohol consumption). Participants were asked to disclose their annual income, in Japanese Yen (1 million Japanese yen equates to approximately 9,000 United States dollars), for the past year according to three categories: ‘less than 2 million’, ‘2–6 million’, and ‘more than 6 million’.

Four health status variables: body mass index (BMI), systolic blood pressure, diabetes, and hypercholesterolemia were also included. Height, weight, HbA1c, blood pressure, and cholesterol were measured by trained clinicians during a health examination. BMI was calculated by dividing weight in kg by height in meters squared and systolic blood pressure was the average of two separate measurements taken after the participant had rested for 5 minutes in a seated position. Diabetes was defined as HbA1c ≥6.5% or receiving anti-diabetic treatment or a diabetes diagnosis. Hypercholesterolemia was defined as having serum total cholesterol level ≥240 mg/dL or receiving lipid lowering medication.

### Statistical analysis

Latent profile analysis (LPA), an advanced person-centered data reduction technique,^19^ was used to identify distinct cluster patterns of the four HRBs, run separately for men and women. LPA aims to identify a categorical latent (ie, unobserved) variable that can explain associations between observed variables in the model. Smoking status and alcohol consumption were treated as ordered variables in the models. To aid model convergence, the number of steps per day and total calorie intake were standardized and included as continuous variables in the models.

LPA models with an increasing number of HRB clusters were compared. Final model selection was based upon the Adjusted Bayesian Information Criterion (aBIC), which balances model fit with parsimony^19^ and adjusts for sample size.^20^ The Lo-Mendell Rubin (LMR) likelihood ratio test was used to determine whether the inclusion of one more cluster improves model fit.^21^ Alongside these fit statistics, model interpretability and cluster size were considered in model selection. Indicator variable specific entropy estimates produced using Mplus Version 8 (Muthen & Muthen, Los Angeles, CA, USA)^22^ were examined, and HRB variables with an entropy <0.2 were removed from the model, as this suggests little contribution in defining the latent HRB clusters.^23^

Once the optimal number of HRB clusters had been identified, gender specific two LPA models were employed in order to account for within-household correlations.^24^

Linear, logistic and multinomial logistic regression models were run in Stata/SE version 16 (Stata Corp, College Station, TX, USA)^25^ to identify associations between HRB cluster membership, socio-demographic variables (age and income), and health status variables (BMI, systolic blood pressure and diabetes diagnosis) while adjusting for age and income. Predicted values and probabilities for socio-demographic and health status variables across the different clusters was calculated using the ‘margins’ postestimation command.

Bivariate analyses using analysis of variance and Chi-square was undertaken to show the means and/or proportions for the socio-demographic and health status variables according to cluster membership. The regression models were adjusted for any classification error that may have occurred during cluster assignment, which prevents under-estimated standard errors of regression coefficients in the regression models.^26^ Missing data was handled using a Full Information Maximum Likelihood Function, under the missing at random (MAR) assumption,^27^ using Mplus version 8.^22^

## RESULTS

### Descriptive analysis

Table 1 describes the HRB, socio-demographic and health status variables.

**Table 1.** Descriptive statistics for Health-related behavior, socio-demographic and health status measures

**Health-related behavior**	**Total**	**Men**	**Women**
	***N* = 8,015 (100%)**	***n* = 3,740 (46.66%)**	***n* = 4,275 (53.34%)**

	**Mean (SD)**	**Mean (SD)**	**Mean (SD)**
Steps per day^a^	6,584.30 (4,220.14)	7,136.93 (4,690.00)	6,117.75 (3,713.42)

Calories per day (kcal)^b^	1,859.04 (554.55)	2,083.80 (584.05)	1,666.65 (445.63)

	***n* (%)**	***n* (%)**	***n* (%)**

Steps *missing*	1,214 (15.13%)	620 (7.72%)	594 (7.46%)

Calories per day (kcal) *missing*	786 (9.87%)	406 (5.19%)	380 (4.72%)

Smoking status^c^			
*Never smoked*	4,487 (55.98%)	1,074 (28.72%)	3,413 (79.84%)
*Former smoker*	1,738 (21.68%)	1,341 (35.86%)	397 (9.29%)
*Current smoker*	1,640 (20.46%)	1,248 (33.37%)	392 (9.71%)
*Missing*	150 (1.87%)	77 (2.06%)	73 (1.71%)

Alcohol^d^			
*None*	3,912 (48.81%)	1,159 (30.99%)	2,753 (64.40%)
*Meets guidelines*	1,011 (12.61%)	403 (10.78%)	608 (14.22%)
*Doesn’t meet guidelines*	2,950 (36.81%)	2,106 (56.31%)	844 (19.74%)
*Missing*	142 (1.77%)	72 (1.93%)	70 (1.64%)

**Socio-demographic and health status**	**Mean (SD)**	**Mean (SD)**	**Mean (SD)**

Age^e^	54.71 (17.76)	53.93 (17.53)	55.40 (17.93)

BMI^f^	22.94 (3.51)	23.57 (3.35)	22.43 (3.56)

Systolic Blood pressure^g^	132.13 (19.59)	136.08 (17.94)	129.35 (20.21)

	***n* (%)**	***n* (%)**	***n* (%)**

Diabetes			
*No*	6,959 (86.82%)	3,113 (83.24%)	3,846 (89.96%)
*Yes*	926 (11.55%)	562 (15.03%)	364 (8.51%)
*missing*	130 (1.62%)	65 (1.74%)	65 (1.52%)

Hypercholesterolemia			
*No*	3,483 (43.46%)	1,580 (42.25%)	1,903 (44.51%)
*Yes*	1,164 (14.52%)	398 (10.64%)	766 (17.92%)
*missing*	3,368 (42.02%)	1,762 (47.11%)	1,606 (37.57%)

Income (Japanese Yen)			
*<2 million*	815 (10.17%)	445 (11.90%)	370 (8.65%)
*2–6 million*	1,964 (24.50%)	1,612 (43.10%)	352 (8.23%)
*>6 million*	721 (9.00%)	648 (17.33%)	73 (1.71%)
*don’t know*	313 (3.91%)	166 (4.44%)	147 (3.44%)
*missing*	4,202 (52.43%)	869 (23.24%)	3,333 (77.96%)

BMI *missing*	1,895 (23.64%)	1,009 (26.98%)	886 (20.73%)

BMI, body mass index; M, mean; SD, standard deviation.

^a^Range, 17–5,001.

^b^Range, 165–5,518.

^c^Current smokers defined as those who have smoked at least 100 cigarettes during their lifetime and smoke daily or on some days. Former smokes do not smoke at all now adays.

^d^According to Japanese government’s alcohol consumption guidelines (≤20 g alcohol per day for men or ≤10 g alcohol per day for women and men aged 65 years or older, 5 days or less a week).

^e^Range, 20–99.

^f^Range, 10.7–41.3.

^g^Range, 81–231.

The sample consisted of 8,015 participants, aged 20 years or over, with slightly more women (53.34%) than men (46.66%). The mean age of the sample was 55 years old (mean 54.71; standard deviation [SD], 17.76 years). Based on the health status measures participants could be considered to be in good health, with average BMI (mean 22.94; SD, 3.51 kg/m^2^), systolic blood pressure readings (mean 132.13; SD, 19.59 mm Hg), and a relatively low proportion defined as having diabetes (*n* = 926, 11.55%) or hypercholesterolemia (*n* = 1,164, 14.52%).

However, more than half of men (*n* = 2,106, 56.31%) exceeded the Japanese government’s recommended levels of alcohol consumption in comparison to just under a fifth of women (*n* = 844, 19.74%). Similarly, a larger proportion of men reported currently smoking (33.4%) in comparison to women (9.2%). On the other hand, a higher number of steps per day were recorded among men (mean 7,136.93; SD, 4,690.00 steps), in comparison to women (mean 6,117.75; SD, 3,713.42 steps). As expected, more daily calories were consumed by men (mean 2,083.80; SD, 584.05 kcal) than women (mean 1,666.65; SD, 445.63 kcal).

### Latent profile analysis

Based on the LPA model fit statistics (see eTable 1), the four cluster model was selected for men and the five cluster model was selected for women. For men, the LMR test was statistically significant in the four cluster model (*P* < 0.001) but not the five cluster model, indicating that the inclusion of a fifth cluster did not improve model fit. For women, while the LMR test was significant for both the fifth and sixth cluster models (*P* < 0.05), the smallest cluster in the sixth cluster was inadequate for further analysis (*n* = 10).

The two-level LPA models, accounting for within-household correlations, further improved model fit for both the four cluster model in men and the five cluster model in women (see eTable 1). Variation in the probability of HRB cluster membership across households was significant for both genders (men *P* = 0.04; women *P* < 0.001).

### Cluster patterns and membership

Estimates from the two-level LPA models are presented in Table 2. For men, the largest cluster (54.28%), labelled ‘Inactive, drinkers’, was characterized by drinking more than Japan’s recommended alcohol guidelines and walking an inadequate number of steps per day. Among women, a similar cluster existed labelled ‘Inactive, drinkers, smokers’, although the size of this cluster was much smaller (16.44%). In contrast, the largest cluster for women (56.58%), labelled ‘Inactive, never smokers, non-drinkers’, was characterized by not smoking or drinking and walking an inadequate number of steps per day. Among men, a slightly smaller cluster (38.24%) with a similar pattern was identified, labelled ‘Inactive, non-drinkers’.

**Table 2.** Estimates for two-level Latent Profile Analysis models, run separately for men and women

	**Men (*N* = 3,740, 100%)**				

	**Cluster 1** ***n* = 1,430** **(38.24%)** *Inactive, non-drinkers*	**Cluster 2** ***n* = 137** **(3.66%)** *Active, drinkers*	**Cluster 3** ***n* = 2,030** **(54.28%)** *Inactive, drinkers*	**Cluster 4** ***n* = 143** **(3.82%)** *Eaters, smokers, drinkers*	

	**Mean (SE)**	**Mean (SE)**	**Mean (SE)**	**Mean (SE)**	

Steps per day	5,694.53 (64.76)	17,402.75 (80.41)	6,125.54 (66.39)	7,451.25 (79.52)	

Calories per day (kcal)	1,691.21 (8.63)	1,945.62 (8.22)	1,875.31 (8.57)	3,011.87 (7.84)	

	**IRP (SE)**	**IRP (SE)**	**IRP (SE)**	**IRP (SE)**	

Smoking status					
*Never smoked*	0.36 (0.01)	0.34 (0.05)	0.20 (0.04)	0.24 (0.06)	
*Former smoker*	0.36 (0.02)	0.28 (0.04)	0.42 (0.02)	0.28 (0.05)	
*Current smoker*	0.28 (0.02)	0.38 (0.05)	0.38 (0.03)	0.48 (0.06)	

Alcohol					
*None*	0.63 (0.15)	0.28 (0.05)	0	0.27 (0.06)	
*Meets guidelines*	0.17 (0.04)	0.07 (0.03)	0.05 (0.04)	0.10 (0.04)	
*Doesn’t meet guidelines*	0.20 (0.17)	0.65 (0.05)	0.95 (0.04)	0.64 (0.06)	

	**Women (*N* = 4,275, 100%)**				
	
	**Cluster 1** ***n* = 703** **(16.44%)** *Inactive, drinkers, smokers*	**Cluster 2** ***n* = 962** **(22.50%)** *Active, moderate drinkers, never smokers*	**Cluster 3** ***n* = 57** **(1.33%)** *Active, mixed drinkers*	**Cluster 4** ***n* = 2,419** **(56.58%)** *Inactive, never smokers, non-drinkers*	**Cluster 5** ***n* = 134** **(3.13%)** *Inactive, eaters, never smokers*
	
	**Mean (SE)**	**Mean (SE)**	**Mean (SE)**	**Mean (SE)**	**Mean (SE)**

Steps per day	5,558.36 (31.35)	10,527.71 (31.98)	20,304.87 (52.63)	4,320.03 (35.90)	6,674.37 (41.73)

Calories per day (kcal)	1,849.84 (5.89)	1,875.49 (6.95)	1,849.16 (7.03)	1,747.94 (7.23)	2,927.42 (5.40)

	**IRP (SE)**	**IRP (SE)**	**IRP (SE)**	**IRP (SE)**	**IRP (SE)**

Smoking status					
*Never smoked*	0.40 (0.19)	0.82 (0.03)	0.62 (0.08)	0.87 (0.01)	0.85 (0.06)
*Former smoker*	0.31 (0.09)	0.10 (0.02)	0.21 (0.07)	0.07 (0.01)	0.08 (0.05)
*Current smoker*	0.29 (0.11)	0.09 (0.02)	0.17 (0.06)	0.06 (0.01)	0.07 (0.04)

Alcohol					
*None*	0	0.61 (0.03)	0.44 (0.08)	0.80 (0.05)	0.53 (0.08)
*Meets guidelines*	0.12 (0.08)	0.18 (0.02)	0.09 (0.04)	0.14 (0.01)	0.16 (0.06)
*Doesn’t meet guidelines*	0.88 (0.08)	0.21 (0.02)	0.47 (0.07)	0.06 (0.05)	0.31 (0.07)

IRP, item response probabilities; M, mean; SE, standard error.

Interestingly, in both genders there was a small cluster (men = 3.66%, women = 1.33%) characterized by a higher than average number of steps per day and a high proportion of members who exceed alcohol consumption guidelines. This cluster was labelled ‘Active, drinkers’ among men and ‘Active, mixed drinkers’ among women.

### Associations between cluster membership and socio-demographic characteristics

Estimates from the regression analyses, adjusting for classification error, are presented in Table 3. These estimates had wider confidence intervals compared to those from models without adjustment (see eTable 2).

**Table 3.** Multinomial logistic regression model estimating association between cluster membership and socio-demographic factors, adjusting for classification error

**Covariates**	**Men sample *N* = 3,740** **Relative risk ratio (95% CI)**				

	**Cluster 1** ***n* = 1,430** **(38.24%)** *Inactive, non-drinkers*	**Cluster 2** ***n* = 137** **(3.66%)** *Active, drinkers*	**Cluster 3** ***n* = 2,030** **(54.28%)** *Inactive, drinkers*	**Cluster 4** ***n* = 143** **(3.82%)** *Eaters, smokers, drinkers*	—

Age	1.02^*^ (1.01 to 1.02)	0.98^*^ (0.97 to 0.99)	*Ref*	0.98^*^ (0.96 to 0.99)	—

Income	0.95 (0.86 to 1.05)	0.76^**^ (0.57 to 0.99)	*Ref*	0.71^**^ (0.51 to 0.99)	—

	**Women sample *N* = 4,275** **Relative risk ratio (95% CI)**				

**Covariates**	**Cluster 1** ***n* = 703** **(16.44%)** *Inactive, drinkers, smokers*	**Cluster 2** ***n* = 962** **(22.50%)** *Active, moderate drinkers, never smokers*	**Cluster 3** ***n* = 57** **(1.33%)** *Active, mixed drinkers*	**Cluster 4** ***n* = 2,419** **(56.58%)** *Inactive, never smokers, non-drinkers*	**Cluster 5** ***n* = 134** **(3.13%)** *Inactive, eaters, never smokers*

Age	0.95^*^ (0.94 to 0.96)	0.97^*^ (0.96 to 0.98)	0.95^*^ (0.92 to 0.97)	*Ref*	0.97^*^ (0.94 to 0.99)

Income	0.98 (0.77 to 1.26)	1.01 (0.86 to 1.17)	0.95 (0.59 to 1.51)	*Ref*	0.83 (0.55 to 1.24)

CI, confidence interval.

^*^P value ≤0.01.

^**^P value ≤0.05.

Associations of HRB cluster membership with socio-demographic variables, age, and income were identified after mutually adjusting for each other in the model. Among men, members of the ‘Inactive, non-drinkers’ cluster were older than those in the reference cluster ‘Inactive, drinkers’. Further bivariate analyses found the mean age of participants in the ‘Inactive, non-drinkers’ cluster to be 55.47 (SD, 19.03) years, higher than the average age in the three remaining clusters, which was 47.52 (SD, 16.20) years, 53.56 (SD, 16.29) years, and 47.21 (SD, 15.82) years, respectively (see eTable 3). Among women, members of the reference cluster ‘Inactive, never smokers, non-drinkers’ were older than those in the remaining four clusters. The mean age of women in each cluster was 45.43 (SD, 15.14) years, 50.71 (SD, 15.35) years, 46.28 (SD, 15.20) years, 59.05 (SD, 18.29) years, and 52.96 (SD, 16.67) years, respectively (see eTable 3).

Male members of the reference cluster ‘Inactive, drinkers’ had a higher annual income in the past year compared to members of the ‘Active drinkers’ and ‘Eaters, smokers, drinkers’ clusters, after adjusting for age. Further bivariate analyses showed over a quarter (*n* = 421, 26.36%) of men in the ‘Inactive, drinkers’ cluster earned more than 6 million Japanese Yen in the past year, compared with 18.06% (*n* = 199), 19.80% (*n* = 20), and 11.27% (*n* = 8) in the remaining three clusters (*P* value <0.05, see eTable 3).

### Associations between cluster membership and health status

**Figure 1.**
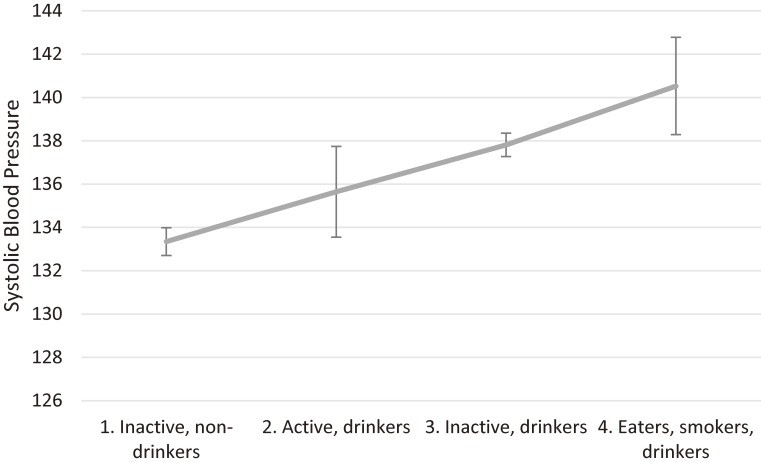
Values of systolic blood pressure by cluster for men, predicted by ordered linear regression analysis adjusting for age, income and classification error, with 95% confidence intervals

Estimates from the regression analyses, adjusting for classification error, are presented in Table 4 and Figure 1. Again, estimate confidence intervals from models without adjustment were narrower (eTable 4).

**Table 4.** Linear and logistic regression models estimating associations between cluster membership and health status, adjusting for age, income and classification error

**Men sample *N* = 3,740**	**BMI**	**Systolic Blood Pressure**	**Diabetes**	**Hypercholesterolemia**

	**Coef (95% CI)** *Constant = 24.12* *Adjusted R2 = 0.01*	**Coef (95% CI)** *Constant = 107.39* *Adjusted R2 = 0.14*	**OR (95% CI)**	**OR (95% CI)**

**Cluster 1** **(*n* = 1,430)** *Inactive, non-drinkers*	Ref	Ref	Ref	Ref

**Cluster 2** **(*n* = 137)** *Active, drinkers*	−0.07 (−0.83 to 0.68)	2.77 (−1.94 to 7.48)	1.03 (0.58 to 1.83)	0.87 (0.44 to 1.73)

**Cluster 3** **(*n* = 2,030)** *Inactive, drinkers*	0.11 (−0.17 to 0.40)	4.86 (3.04 to 6.68)^*^	0.94 (0.76 to 1.15)	0.99 (0.77 to 1.27)

**Cluster 4** **(*n* = 143)** *Eaters, smokers, drinkers*	1.20 (0.36 to 2.05)^*^	7.78 (2.48 to 13.09)^*^	0.52 (0.22 to 1.23)	0.64 (0.26 to 1.55)

**Age**	−0.01 (−0.02 to −0.01)^**^	0.46 (0.40 to 0.53)^*^	1.04 (1.03 to 1.05)^*^	1.02 (1.01 to 1.02)^*^

**Income**	0.03 (−0.16 to 0.21)	−0.88 (−2.08 to 0.32)	0.87 (0.76 to 0.99)^**^	1.03 (0.88 to 1.22)

**Women sample *N* = 4,275**	**BMI**	**Systolic Blood Pressure**	**Diabetes**	**Hypercholesterolemia**

	**Coef (95% CI)** *Constant = 20.76* *Adjusted R2 = 0.02*	**Coef (95% CI)** *Constant = 94.34* *Adjusted R2 = 0.25*	**OR (95% CI)**	**OR (95% CI)**

**Cluster 1** **(*n* = 703)** *Inactive, drinkers, smokers*	Ref	Ref	Ref	Ref

**Cluster 2** **(*n* = 962)** *Active, moderate drinkers, non-smokers*	−0.15 (−1.15 to 0.84)	−0.26 (−6.40 to 5.87)	0.88 (0.36 to 2.20)	1.67 (0.78 to 3.56)

**Cluster 3** **(*n* = 57)** *Active, mixed drinkers*	0.46 (−1.52 to 2.44)	6.60 (−5.81 to 19.01)	1.99 (0.45 to 8.89)	3.92 (0.99 to 15.52)

**Cluster 4** **(*n* = 2,419)** *Inactive, non-smokers, non-drinkers*	0.25 (−0.69 to 1.19)	0.82 (−5.02 to 6.66)	0.89 (0.38 to 2.07)	1.38 (0.67 to 2.86)

**Cluster 5** **(*n* = 134)** *Inactive, eaters, non-smokers*	−0.37 (−1.98 to 1.24)	0.05 (−9.08 to 9.19)	0.57 (0.11 to 2.98)	1.01 (0.32 to 3.21)

**Age**	0.03 (0.02 to 0.05)^*^	0.67 (0.57 to 0.77)^*^	1.04 (1.03 to 1.06)^*^	1.03 (1.01 to 1.04)^*^

**Income**	−0.03 (−0.27 to 0.20)	−1.41 (−2.83 to 0.01)	0.90 (0.74 to 1.09)	0.92 (0.78 to 1.08)

CI, confidence interval; Coef, linear regression coefficient; OR, logistic regression odds ratio.

^*^ = *P* value <0.01.

^**^ = *P* value ⩽0.05.

Among men, members of the ‘Eaters, smokers, drinkers’ cluster had a higher BMI than the reference cluster ‘Inactive, non-drinkers’, after adjusting for age and income. The mean BMI in the ‘Eaters, smokers, drinkers’ cluster was 25.09 (SD, 4.08) kg/m^2^, higher than the other three clusters, which were 23.41 (SD, 3.47) kg/m^2^, 23.12 (SD, 3.04) kg/m^2^, and 23.62 (SD, 3.20) kg/m^2^, respectively, and sufficiently high to cross the threshold for the definition of overweight (see eTable 5). No significant association between HRB cluster membership and BMI was found for women after adjusting for age and income.

Similarly, after adjusting for age and income, systolic blood pressure was higher among men in the ‘Inactive, drinkers’ and ‘Eaters, smokers, drinkers’ clusters compared to the reference cluster ‘Inactive, non-drinkers’. The mean systolic blood pressure in these two clusters were 137.44 (SD, 17.86) mm Hg and 137.28 (SD, 19.86) mm Hg, respectively. In comparison to the other two clusters, which were 134.43 (SD, 17.80) mm Hg and 132.46 (SD, 17.50) mm Hg, respectively (see eTable 5). There were no significant associations between systolic blood pressure and HRB cluster membership among women.

Finally, no significant association was identified between HRB cluster membership and diabetes or hypercholesterolemia in either gender, after adjusting for age and income.

## DISCUSSION

This study is the first to investigate the clustering of four HRBs (ie, smoking, alcohol, diet, and physical activity) in a representative sample of the adult Japanese population. Using latent variable modelling we have identified four male and five female distinct patterns of HRBs, whose membership is associated to varying degrees with both socio-demographic characteristics (ie, age and income) and health status (ie, BMI, systolic blood pressure, diabetes, and hypercholesterolemia).

The finding that HRB clusters were distinguishable from one another largely due to differences in alcohol consumption and physical activity, particularly among men, is not surprising given the evidence of a complex relationship between these two behaviors.^28^ Moreover, the largest cluster among women was characterized by never smokers and non-drinkers, whereas the largest cluster among men had a very high probability (95%) of exceeding the Japanese government’s alcohol consumption guidelines. This finding is in keeping with evidence of large gender differences in alcohol consumption in Japan.^29^

The average income for men in the largest cluster, ‘Inactive, drinkers’, was higher than that of those in the other three, which supports existing evidence of a relationship between higher income and work-related drinking which is commonly practiced among Japanese men.^30^

Many of the HRB clusters for men and women were characterized by low levels of physical activity. Evidence suggest that the average number of steps among Japanese adults has decreased since 2000.^31^ This lack of exercise has subsequently led to the release of “+10 minutes of physical activity per day” recommendations by the Japanese government.^32^ Such interventions may be more complex if the low levels of physical activity are related to high levels of drinking and smoking and may necessitate more complex designs aimed at simultaneously reducing smoking and drinking or finding lifestyle recommendations that involve replacing alcohol-related lifestyle choices (such as regular after-work drinking) with healthier activities.

Men in clusters characterized by more negative HRBs, namely the ‘Inactive, drinkers’ and ‘Eaters, smokers, drinkers’ clusters, had higher systolic blood pressure than the other two. This is likely due to the propensity towards heavy drinking in these two clusters.^33^ Furthermore, men in the latter cluster, whose average calorie intake was over 30% more than that of the other clusters, also had a higher BMI than men in the other three clusters. Curiously, there was no relationship between health status and HRB cluster membership among women, despite the presence of a cluster characterized by multiple negative HRBs, namely ‘Inactive, drinkers, smokers’. This may be, in part, due to the lower proportion of current smokers in this cluster, when compared to men. Moreover, while the probability of women exceeding alcohol consumption guidelines in this cluster is high compared to women in the remaining cluster, it could be that consumption is still rather low when compared to men. As expected, research has found greater care-giving responsibilities is associated with lower levels of drinking among women.^34^ It is therefore likely that, even among women who do drink heavily in this study, the majority will have greater domestic responsibilities and therefore less opportunities to drink heavily than their male counterparts.

The lack of association between HRB cluster membership and diabetes or hypercholesterolemia could be due to the relatively young sample in this study (mean age 54.71; SD, 17.76 years), but might also be due to the very low prevalence of diabetes and hypercholesterolemia in the Japanese population.^35^^,^^36^ Longitudinal data that can investigate the association between mid-life HRB cluster membership and older age-related chronic diseases such as diabetes, heart disease and cancer would be a fruitful area of future research.

### Strengths and limitations

A strength of this study is its novel approach to investigating lifestyles in the Japanese population, focusing on how HRBs inter-related with one another rather than treating them as individual entities. The NHNS data is a powerful resource, being the only Japanese health examination and interview study conducted at a national level.^15^ Access to objectively measured HRB and health status measures (ie, steps per day, systolic blood pressure, and BMI) removes reporting and social desirability bias. Moreover, measuring total calorie intake in a 24-hour period minimizes recall bias which is an inherent limitation of most dietary measures.^37^

While the NHNS survey data is mostly representative of the Japanese population, it is acknowledged that some groups are underrepresented. For example, the 2010 survey includes 6% of adults who are living in single-person households, which is 9% lower than national estimates.^15^ The cross-sectional survey design, while useful to identify trends at a population level over time, is less useful in tracking longer term health outcomes associated with lifestyles (ie, diabetes, heart disease, cancer).

Furthermore, while the objective and short-term measures of HRBs are useful in minimizing certain types of bias, they may not be a true representation of each participant’s everyday habits. Given these limitations, it is important to replicate this research using other HRBs and study designs (eg, cohort studies, routine administrative data) in order to triangulate the study findings suffering from different types of bias and reaching consensus.^38^

### Public health implications

The results of this study indicate a complex inter-relationship between HRBs, which are differentially associated with gender, socio-demographic factors, and health status. By identifying these characteristics of HRB cluster membership we have a better understanding of the types of people who share particular lifestyles. This may inform the development of more sophisticated, holistic and person-centered approaches, which can target specific subgroups of the population and effectively tackle negative HRBs in Japan and other high-income countries.^2^ For example, the results of this work support the findings of a study conducted in the United Kingdom, which identified a small cluster characterized by heavy smoking and physical inactivity and whose participants were more likely to be overweight/obese and in a more disadvantaged socio-economic position.^39^

The association between higher systolic blood pressure and male members of the largest cluster, ‘Inactive, drinkers’, characterized by heavy drinking and physical inactivity, is a pressing concern. Given that members of this cluster also have a higher income than other clusters, aspects of the male-dominated white collar Japanese workplace culture, often denoted as the smoking and drinking, hard-working and hard-playing ‘salaryman’,^40^ may have long-term negative health consequences for these men.

### Conclusion

This detailed investigation of HRB clustering within the Japanese population addresses an important gap in the evidence base. Detecting distinct clusters of HRBs in a Japanese population-based survey provides a person-centered understanding of Japanese lifestyles that can inform population level interventions to improve HRBs among Japanese adults. Understanding how HRBs cluster in the Japanese population can also assist overseas policy makers to identify new strategies for targeting behavioral risk factors and make health promotion policies more effective.

## APPENDIX A. SUPPLEMENTARY DATA

eTable 1. Latent Profile Analysis model fit statistics

eTable 2. Multinomial logistic regression model estimating association between cluster membership and socio-demographic factors (no adjustment for classification error)

eTable 3. Bivariate analyses of socio-demographic variables and cluster membership

eTable 4. Linear and logistic regression models estimating associations between cluster membership and health status, adjusting for age and income (no adjustment for classification error)

eTable 5. Bivariate analyses^a^ of health status variables and cluster membership

## References

[1] Meader N, King K, Moe-Byrne T, . A systematic review on the clustering and co-occurrence of multiple risk behaviours. BMC Public Health. 2016 Dec;16(1):657. 10.1186/s12889-016-3373-6 27473458 PMC4966774

[2] Noble N, Paul C, Turon H, Oldmeadow C. Which modifiable health risk behaviours are related? A systematic review of the clustering of Smoking, Nutrition, Alcohol and Physical activity (‘SNAP’) health risk factors. Prev Med. 2015 Dec;81:16–41. 10.1016/j.ypmed.2015.07.003 26190368

[3] Cockerham WC, Wolfe JD, Bauldry S. Health Lifestyles in Late Middle Age. Res Aging. 2020 Jan;42(1):34–46. 10.1177/0164027519884760 31709904

[4] Filippidis FT, Agaku IT, Vardavas CI. Geographic variation and socio-demographic determinants of the co-occurrence of risky health behaviours in 27 European Union member states. J Public Health (Oxf). 2016 Jun 1;38(2):e13–e20. 10.1093/pubmed/fdv061 25968134

[5] Graham H, Hutchinson J, Law C, Platt L, Wardle H. Multiple health behaviours among mothers and partners in England: clustering, social patterning and intra-couple concordance. SSM Popul Health. 2016 Dec;2:824–833. 10.1016/j.ssmph.2016.10.011 28018962 PMC5165044

[6] Charvat H, Sasazuki S, Inoue M, ; JPHC Study Group. Impact of five modifiable lifestyle habits on the probability of cancer occurrence in a Japanese population-based cohort: results from the JPHC study. Prev Med. 2013;57(5):685–689. 10.1016/j.ypmed.2013.08.030 24021992

[7] Tanaka H, Sasazawa Y, Suzuki S, Nakazawa M, Koyama H. Health status and lifestyle factors as predictors of depression in middle-aged and elderly Japanese adults: a seven-year follow-up of the Komo-Ise cohort study. BMC Psychiatry. 2011 Dec;11(1):20. 10.1186/1471-244X-11-20 21294921 PMC3041738

[8] Sasazuki S, Inoue M, Iwasaki M, ; JPHC Study Group. Combined impact of five lifestyle factors and subsequent risk of cancer: the Japan Public Health Center Study. Prev Med. 2012;54(2):112–116. 10.1016/j.ypmed.2011.11.003 22155160

[9] World Health Organization. Non-communicable diseases country profiles 2018 [Internet]. Geneva: World Health Organization; 2018. Available from: https://www.who.int/nmh/publications/ncd-profiles-2018/en/.

[10] Lahelma E, Lallukka T, Laaksonen M, . Social class differences in health behaviours among employees from Britain, Finland and Japan: the influence of psychosocial factors. Health Place. 2010 Jan;16(1):61–70. 10.1016/j.healthplace.2009.08.004 19762272

[11] Ng M, Freeman MK, Fleming TD, . Smoking prevalence and cigarette consumption in 187 countries, 1980–2012. JAMA. 2014;311(2):183–192. 10.1001/jama.2013.284692 24399557

[12] Anderson CAM, Appel LJ, Okuda N, . Dietary sources of sodium in China, Japan, the United Kingdom, and the United States, women and men aged 40 to 59 years: the INTERMAP study. J Am Diet Assoc. 2010 May;110(5):736–745. 10.1016/j.jada.2010.02.007 20430135 PMC4308093

[13] Ng M, Fleming T, Robinson M, . Global, regional, and national prevalence of overweight and obesity in children and adults during 1980–2013: a systematic analysis for the Global Burden of Disease Study 2013. Lancet. 2014 Aug;384(9945):766–781. 10.1016/S0140-6736(14)60460-8 24880830 PMC4624264

[14] Nomura S, Sakamoto H, Glenn S, . Population health and regional variations of disease burden in Japan, 1990–2015: a systematic subnational analysis for the Global Burden of Disease Study 2015. Lancet. 2017 Sep;390(10101):1521–1538. 10.1016/S0140-6736(17)31544-1 28734670 PMC5613077

[15] Ikeda N, Takimoto H, Imai S, Miyachi M, Nishi N. Data resource profile: the Japan National Health and Nutrition Survey (NHNS). Int J Epidemiol. 2015 Dec 1;44(6):1842–1849. 10.1093/ije/dyv152 26239276

[16] Katanoda K, Matsumura Y. National nutrition survey in Japan. Its methodological transition and current findings. J Nutr Sci Vitaminol (Tokyo). 2002;48(5):423–432. 10.3177/jnsv.48.423 12656220

[17] Katanoda K, Nitta H, Hayashi K, Matsumura Y. Is the national nutrition survey in Japan representative of the entire Japanese population? Nutrition. 2005 Sep;21(9):964–966. 10.1016/j.nut.2005.02.004 16039832

[18] International Alliance for Responsible Drinking. Drinking guidelines: General population [Internet]. Drinking guidelines: General population. 2019. Available from: https://iard.org/science-resources/detail/Drinking-Guidelines-General-Population.

[19] Collins LM, Lanza ST. Latent Class and Latent Transition Analysis [Internet]. Hoboken, NJ, USA: John Wiley & Sons, Inc.; 2009 [cited 2020 Feb 19]. (Wiley Series in Probability and Statistics). Available from: http://doi.wiley.com/10.1002/9780470567333.

[20] Finch H. A comparison of statistics for assessing model invariance in latent class analysis. OJS. 2015;05(03):191–210. 10.4236/ojs.2015.53022

[21] Nylund KL, Asparouhov T, Muthén BO. Deciding on the number of classes in latent class analysis and growth mixture modeling: a Monte Carlo simulation study. Struct Equ Modeling. 2007 Oct 23;14(4):535–569. 10.1080/10705510701575396

[22] Muthén M. Mplus [Internet]. Los Angeles, CA: Muthén & Muthén; 2017. Available from: https://www.statmodel.com/.

[23] Asparouhov T, Muthén B. Auxiliary variables in mixture modeling: three-step approaches using M *plus*. Struct Equ Modeling. 2014 Jul 3;21(3):329–341. 10.1080/10705511.2014.915181

[24] Muthén L, Muthén B. Mplus User’s Guide. 8th Edition. [Internet]. Los Angeles, CA: Muthén & Muthén; 2017. Available from: https://www.statmodel.com/ugexcerpts.shtml.

[25] StataCorp. Stata Statistical Software [Internet]. Texas, USA: StataCorp; 2019. Available from: https://www.stata.com/company/.

[26] Clark S, Muthén B. Relating Latent Class Analysis Results to Variables not Included in the Analysis [Internet]. statmodel; 2009. Available from: https://www.statmodel.com/download/relatinglca.pdf.

[27] Enders CK. *Applied missing data analysis*. New York: Guilford Press; 2010. 377 p. (Methodology in the social sciences).

[28] Leasure JL, Neighbors C, Henderson CE, Young CM. Exercise and Alcohol Consumption: What We Know, What We Need to Know, and Why it is Important. Front Psychiatry [Internet]. 2015 Nov 2 [cited 2020 Feb 19];6. Available from: http://journal.frontiersin.org/Article/10.3389/fpsyt.2015.00156/abstract. 10.3389/fpsyt.2015.00156 PMC4629692 26578988

[29] World Health Organization. Global status report on alcohol and health 2018 [Internet]. Geneva: World Health Organization; 2018. Available from: https://www.who.int/substance_abuse/publications/global_alcohol_report/en/.

[30] Murakami K, Hashimoto H. Associations of education and income with heavy drinking and problem drinking among men: evidence from a population-based study in Japan. BMC Public Health. 2019 Dec;19(1):420. 10.1186/s12889-019-6790-5 31014312 PMC6480518

[31] Tanaka S. Status of physical activity in Japanese adults and children. Ann Hum Biol. 2019 May 19;46(4):305–310. 10.1080/03014460.2019.1635644 31234661

[32] Miyachi M, Tripette J, Kawakami R, Murakami H. +10 min of Physical Activity per day: Japan is looking for efficient but feasible recommendations for its population. J Nutr Sci Vitaminol (Tokyo). 2015;61(Suppl):S7–S9. 10.3177/jnsv.61.S7 26598893

[33] Roerecke M, Kaczorowski J, Tobe SW, Gmel G, Hasan OSM, Rehm J. The effect of a reduction in alcohol consumption on blood pressure: a systematic review and meta-analysis. The Lancet Public Health. 2017 Feb;2(2):e108–e120. 10.1016/S2468-2667(17)30003-8 29253389 PMC6118407

[34] Takeda Y, Kawachi I, Yamagata Z, . The impact of multiple role occupancy on health-related behaviours in Japan: differences by gender and age. Public Health. 2006 Oct;120(10):966–975. 10.1016/j.puhe.2006.06.006 16949626

[35] Ogushi Y, Hamazaki T, Kirihara Y. Blood Cholesterol as a Good Marker of Health in Japan. In: Simopoulos AP, De Meester F, editors. World Review of Nutrition and Dietetics [Internet]. Basel: KARGER; 2009 [cited 2020 May 4]. p. 63–70. Available from: https://www.karger.com/Article/FullText/235712. 10.1159/000235712 19696528

[36] Ma D, Sakai H, Wakabayashi C, . The prevalence and risk factor control associated with noncommunicable diseases in China, Japan, and Korea. J Epidemiol. 2017 Dec;27(12):568–573. 10.1016/j.je.2016.12.019 28623056 PMC5623033

[37] Althubaiti A. Information bias in health research: definition, pitfalls, and adjustment methods. J Multidiscip Healthc. 2016;9:211–217. 10.2147/JMDH.S104807 27217764 PMC4862344

[38] Lawlor DA, Tilling K, Davey Smith G. Triangulation in aetiological epidemiology. Int J Epidemiol. 2016;45(6):1866–1886. 10.1093/ije/dyw314 28108528 PMC5841843

[39] Birch J, Petty R, Hooper L, Bauld L, Rosenberg G, Vohra J. Clustering of behavioural risk factors for health in UK adults in 2016: a cross-sectional survey. J Public Health (Oxf). 2019 Sep 30;41(3):e226–e236. 10.1093/pubmed/fdy144 30192965 PMC6785700

[40] Alexander JW. Medicating the salaryman lifestyle: fear-based marketing of liver stimulant drugs in postwar Japan. Japan Forum. 2015 Apr 3;27(2):134–166. 10.1080/09555803.2015.1040819

