# Supplementary material for: The Clustering of Health-Related Behaviors in the Adult Japanese Population
Source: J Epidemiol. 2021 Aug 5;31(8):471–9. doi: 10.2188/jea.JE20200120 (PMC8275444; doi:10.2188/jea.JE20200120)
Supplement: Supplementary file 1 [file je-31-471-s001.pdf]

eTable 1. Latent Profile Analysis model fit statistics

Men (N=3,740)	Log-likelihood	aBIC	LMR	Smallest cluster (n)
2 cluster	-16230.54	32536.81	0.01	158
3 cluster	-16123.12	32357.32	0.01	141
**4 cluster**	**-16054.32**	**32255.08**	**<0.01**	**103**
5 cluster	-16004.84	32191.45	0.47	4
**Two-level 4 cluster**	**-16022.49**	**32206.55**	**<0.01**	**137**
**Women (N=4,275)**	**Log-likelihood**	**aBIC**	**LMR**	**Smallest cluster (n)**
2 cluster	-16955.27	33988.28	0.01	202
3 cluster	-16842.74	33799.50	<0.01	143
4 cluster	-16770.39	33691.09	0.04	63
**5 cluster**	**-16719.73**	**33626.05**	**0.01**	**61**
6 cluster	-16684.41	33591.69	0.03	10
7 cluster	-16661.57	33582.28	0.85	5
**Two-level 5 cluster**	**-16687.81**	**33582.93**	**0.01**	**57**

aBIC, adjusted Bayesian Information Criterion; *LMR, Lo-Mendell Rubin likelihood ratio test.*

eTable 2. Multinomial logistic regression model estimating association between cluster membership and socio-demographic factors (no adjustment for classification error)

**Covariates**	**Men sample N=3,740**<br>**Relative risk ratio (95% CI)**				
	**Cluster 1**<br>**n=1,430 (38.24%)**<br>*Inactive, non-drinkers*	**Cluster 2**<br>**n=137 (3.66%)**<br>*Active, drinkers*	**Cluster 3**<br>**n=2,030 (54.28%)**<br>*Inactive, drinkers*	**Cluster 4**<br>**n=143 (3.82%)**<br>*Eaters, smokers, drinkers*	**-**
Age	1.02*<br>(1.01 to 1.01)	0.98*<br>(0.98 to 0.98)	*Ref*	0.98*<br>(0.98 to 0.98)	-
Income	0.95<br>(0.92 to 0.98)*	0.76*<br>(0.69 to 0.84)	*Ref*	0.71*<br>(0.64 to 0.80)	-
	**Women sample N=4,275**<br>**Relative risk ratio (95% CI)**				
**Covariates**	**Cluster 1**<br>**n=703 (16.44%)**<br>*Inactive, drinkers, smokers*	**Cluster 2**<br>**n=962 (22.50%)**<br>*Active, moderate drinkers, never smokers*	**Cluster 3**<br>**n=57 (1.33%)**<br>*Active, mixed drinkers*	**Cluster 4**<br>**n=2,419 (56.58%)**<br>*Inactive, never smokers, non-drinkers*	**Cluster 5**<br>**n=134 (3.13%)**<br>*Inactive, eaters, never smokers*
Age	0.95*<br>(0.95 to 0.96)	0.97*<br>(0.97 to 0.97)	0.95*<br>(0.95 to 0.96)	*Ref*	0.98*<br>(0.98 to 0.98)
Income	0.98<br>(0.91 to 1.07)	1.01<br>(0.96 to 1.06)	0.95<br>(0.81 to 1.11)	*Ref*	0.83*<br>(0.72 to 0.95)

CI, confidence interval.
* p value <0.01.

eTable 3. Bivariate analyses of socio-demographic variables and cluster membership

**Men**	**Cluster 1** *Inactive, non-drinkers*	**Cluster 2** *Active, drinkers*	**Cluster 3** *Inactive, drinkers*	**Cluster 4** *Eaters, smokers, drinkers*	-
**Age** *Mean (SD)**	55.47 (19.03)	47.52 (16.20)	53.56 (16.29)	47.21 (15.82)	-
**Income** *n (%)**<br>* *<2 million yen*<br>*2-6 million yen*<br>*>6 million yen*<br>*Don't know*	 207 (18.78%)<br>609 (55.26%)<br>199 (18.06%)<br>87 (7.89%)	 15 (14.85%)<br>63 (62.38%)<br>20 (19.80%)<br>3 (2.97%)	 215 (13.46%)<br>888 (55.60%)<br>421 (26.36%)<br>73 (4.57%)	 8 (11.27%)<br>52 (73.24%)<br>8 (11.27%)<br>3 (4.23%)	-
**Women**	**Cluster 1** *Inactive, drinkers, smokers*	**Cluster 2** *Active, moderate drinkers, never smokers*	**Cluster 3** *Active, mixed drinkers*	**Cluster 4** *Inactive, never smokers, non-drinkers*	**Cluster 5** *Inactive, eaters, never smokers*
**Age** *Mean (SD)**	45.43 (15.14)	50.71 (15.35)	46.28 (15.20)	59.05 (18.29)	52.96 (16.67)
**Income** *n (%)*<br>*<2 million yen*<br>*2-6 million yen*<br>*>6 million yen*<br>*Don't know*	 28 (38.36%)<br>26 (35.62%)<br>4 (5.48%)<br>15 (20.55%)	 72 (31.30%)<br>104 (45.22%)<br>24 (10.43%)<br>30 (13.04%)	 5 (26.32%)<br>10 (52.63%)<br>3 (10.53%)<br>2 (10.53%)	 254 (42.91%)<br>201 (33.95%)<br>38 (6.42%)<br>99 (16.72%)	 11 (39.29%)<br>11 (39.29%)<br>5 (17.86%)<br>1 (3.57%)

M, mean; SD, standard deviation.

* p value <0.01 one-way ANOVA.

** p value ≤0.05 Chi-square.

eTable 4. Linear and logistic regression models estimating associations between cluster membership and health status, adjusting for age and income (no adjustment for classification error)

Men sample N=3,740	BMI	Systolic Blood Pressure	Diabetes	Hypercholesterolemia
	**Coef (95% CI)** [ab]<br>*Constant= 24.12*<br>*Adjusted R2= 0.01*	**Coef (95% CI)**<br>*Constant= 107.39*<br>*Adjusted R2= 0.14*	**OR (95% CI)** [c]	**OR (95% CI)**
**Cluster 1**<br>**(n=1,430)**<br>*Inactive, non-drinkers*	Ref	Ref	Ref	Ref
**Cluster 2**<br>**(n=137)**<br>*Active, drinkers*	-0.07 (-0.33 to 0.18)	2.77 (1.20 to 4.33)*	1.03 (0.86 to 1.25)	0.87 (0.69 to 1.09)
**Cluster 3**<br>**(n=2,030)**<br>*Inactive, drinkers*	0.11 (0.02 to 0.21)** [e]	4.86 (4.26 to 5.47)*	0.94 (0.88 to 1.01)	0.99 (0.91 to 1.08)
**Cluster 4**<br>**(n=143)**<br>*Eaters, smokers, drinkers*	1.20 (0.92 to 1.48)* [d]	7.78 (6.02 to 9.55)*	0.52 (0.39 to 0.69)*	0.64 (0.47 to 0.86)*
**Age**	-0.01 (-0.01 to -0.01)*	0.46 (0.44 to 0.49)*	1.04 (1.04 to 1.04)*	1.02 (1.01 to 1.02)*
**Income**	0.03 (-0.03 to 0.09)	-0.88 (-1.28 to -0.48)*	0.87 (0.83 to 0.91)*	1.03 (0.98 to 1.09)
**Women sample N=4,275**	**BMI**	**Systolic Blood Pressure**	**Diabetes**	**Hypercholesterolemia**
	**Coef (95% CI)**<br>*Constant= 20.76*<br>*Adjusted R2= 0.02*	**Coef (95% CI)**<br>*Constant= 94.34*<br>*Adjusted R2= 0.25*	**OR (95% CI)**	**OR (95% CI)**
**Cluster 1**<br>**(n=703)**<br>*Inactive, drinkers, smokers*	Ref	Ref	Ref	Ref
**Cluster 2**<br>**(n=962)**<br>*Active, moderate drinkers, non-smokers*	-0.15 (-0.48 to 0.18)	-0.26 (-2.29 to 1.77)	0.88 (0.65 to 1.20)	1.67 (1.30 to 2.15)*
**Cluster 3**<br>**(n=57)**<br>*Active, mixed drinkers*	0.46 (-0.20 to 1.12)	6.60 (2.50 to 10.70)*	1.99 (1.21 to 3.28)*	3.92 (2.48 to 6.20)*
**Cluster 4**<br>**(n=2,419)**<br>*Inactive, non-smokers, non-drinkers*	0.25 (-0.06 to 0.56)	0.82 (-1.12 to 2.75)	0.89 (0.67 to 1.18)	1.38 (1.09 to 1.76)*
**Cluster 5**<br>**(n=134)**<br>*Inactive, eaters, non-smokers*	-0.37 (-0.90 to 0.17)	0.05 (-2.97 to 3.08)	0.57 (0.32 to 0.98)**	1.01 (0.69 to 1.48)
**Age**	0.03 (0.03 to 0.04)*	0.67 (0.64 to 0.70)*	1.04 (1.04 to 1.05)*	1.03 (1.02 to 1.03)*
**Income**	-0.03 (-0.11 to 0.05)	-1.41 (-1.88 to -0.94)*	0.90 (0.85 to 0.96)*	0.92 (0.87 to 0.97)*

CI, confidence interval; Coef, Linear regression coefficient; OR, logistic regression odds ratio.

* p value <0.01.

** p value ≤0.05.

eTable 5. Bivariate analyses[a] of health status variables and cluster membership

**Men**	**Cluster 1** *Inactive, non-drinkers*	**Cluster 2** *Active, drinkers*	**Cluster 3** *Inactive, drinkers*	**Cluster 4** *Eaters, smokers, drinkers*	-
**BMI** *Mean (SD)**	23.41 (3.47)	23.12 (3.04)	23.62 (3.20)	25.09 (4.08)	-
**Systolic Blood Pressure** *Mean (SD)**	134.43 (17.80)	132.46 (17.50)	137.44 (17.86)	137.28 (19.86)	
**Diabetes Diagnosis** *n (%)***<br>*No*<br>*Yes*	 1,231 (82.90)<br>254 (17.10)	 119 (86.23)<br>19 (13.77)	 1,668 (85.54)<br>282 (14.46)	 95 (93.14)<br>7 (6.86)	-
**Women**	**Cluster 1** *Inactive, drinkers, smokers*	**Cluster 2** *Active, moderate drinkers, never smokers*	**Cluster 3** *Active, mixed drinkers*	**Cluster 4** *Inactive, never smokers, non-drinkers*	**Cluster 5** *Inactive, eaters, never smokers*
**BMI** *Mean (SD)**	21.98 (3.50)	22.05 (3.30)	21.37 (3.30)	22.69 (3.68)	22.23 (3.20)
**Systolic Blood Pressure** *Mean (SD)**	121.85 (18.29)	126.81 (18.98)	125.30 (18.42)	131.69 (20.69)	128.98 (19.18)
**Diabetes Diagnosis** *n (%)***<br>*No*<br>*Yes*	 376 (96.16)<br>15 (3.84)	 966 (92.09)<br>83 (7.91)	 56 (91.80)<br>5 (8.20)	 2,347 (90.27)<br>253 (9.73)	 101 (92.66)<br>8 (7.34)

M, mean; SD, standard deviation.

[a] No adjustment for age or income.

* p value <0.01 one-way ANOVA.

** p value ⩽0.05 Chi-square.
